# Breathing Rhythm Variations during Wash-In Do Not Influence Exhaled Volatile Organic Compound Profile Analyzed by an Electronic Nose

**DOI:** 10.3390/molecules26092695

**Published:** 2021-05-04

**Authors:** Silvano Dragonieri, Vitaliano Nicola Quaranta, Pierluigi Carratù, Teresa Ranieri, Enrico Buonamico, Giovanna Elisiana Carpagnano

**Affiliations:** 1 Respiratory Diseases, University of Bari, 70121 Bari, Italy; teresa.ranieri@uniba.it (T.R.); enricobuonamico@gmail.com (E.B.); elisiana.carpagnano@uniba.it (G.E.C.); 2 Pulmonology, Di Venere Hospital, 70131 Bari, Italy; vitalianonicola.40@gmail.com; 3 Internal Medicine “A. Murri”, University of Bari, 70121 Bari, Italy; pierluigi.carratu@uniba.it

**Keywords:** volatile organic compounds, e-nose, electronic nose, breath analysis, breathing rhythm

## Abstract

E-noses are innovative tools used for exhaled volatile organic compound (VOC) analysis, which have shown their potential in several diseases. Before obtaining a full validation of these instruments in clinical settings, a number of methodological issues still have to be established. We aimed to assess whether variations in breathing rhythm during wash-in with VOC-filtered air before exhaled air collection reflect changes in the exhaled VOC profile when analyzed by an e-nose (Cyranose 320). We enrolled 20 normal subjects and randomly collected their exhaled breath at three different breathing rhythms during wash-in: (a) normal rhythm (respiratory rate (RR) between 12 and 18/min), (b) fast rhythm (RR > 25/min) and (c) slow rhythm (RR < 10/min). Exhaled breath was collected by a previously validated method (Dragonieri et al., J. Bras. Pneumol. 2016) and analyzed by the e-nose. Using principal component analysis (PCA), no significant variations in the exhaled VOC profile were shown among the three breathing rhythms. Subsequent linear discriminant analysis (LDA) confirmed the above findings, with a cross-validated accuracy of 45% (*p* = ns). We concluded that the exhaled VOC profile, analyzed by an e-nose, is not influenced by variations in breathing rhythm during wash-in.

## 1. Introduction

The recent evolutions in sensor manufacturing and software advances have generated new promising devices for detecting and quantifying the numerous volatile organic compounds (VOCs) which originate from our metabolism [1]. Among these instruments, electronic noses (e-noses) imitate mammalian olfaction in order to obtain reproducible measurements of VOC profiles in human mediums such as urine, blood, or breath [2]. In addition, exhaled breath analysis by e-nose can be used as a noninvasive biomarker of various metabolic pathways occurring in health and illness. Interestingly, an increasing number of studies have revealed the potential for the application of VOC profiling in numerous respiratory and systemic diseases [3].

Recently, a European Respiratory Society (ERS) task force document established guidelines in order to standardize all the methodological concerns for breath sampling and analysis by e-noses [4]. In these guidelines, it is unmistakably indicated that, when investigating exhaled VOCs, non-disease, patient-related factors, such as breathing manoeuvers, airway caliber, food and beverages intake, physical exercise and pregnancy, should always be considered [4].

Among these, intra-/inter-individual subjects’ own respiratory physiology-associated variations may represent important confounders in exhaled VOC profiling [5,6]. In particular, the conditioning of inspiratory air and the expiratory breathing maneuvers may both influence the VOC pattern [7]. Indeed, the control of breathing is mainly automatic, and its regulation is driven by the autonomic nervous regulation of the respiratory center in the human brain [8]. Therefore, modifications of ventilatory patterns may result in exhaled alveolar concentrations of VOCs, the exhalations of which are dependent on minute ventilation and/or on CO2 exhalation. Moreover, ventilatory variations are known to modify arterial CO2 pressure levels, cardiac output and pulse pressure in humans [8,9]. Although a 5 min steady-state washing-in with VOC-filtered air is suggested, based on recommendations for helium washing during lung volume measurements, it is not clear whether it needs to be modified in certain types of patients.

For the above reason, the aim of the current study was to assess whether variations in breathing rhythms during the wash-in phase reflect changes in the exhaled VOC profile when analyzed by an e-nose.

## 2. Results

The characteristics of the study population are described in Table 1.

**Figure 1:**
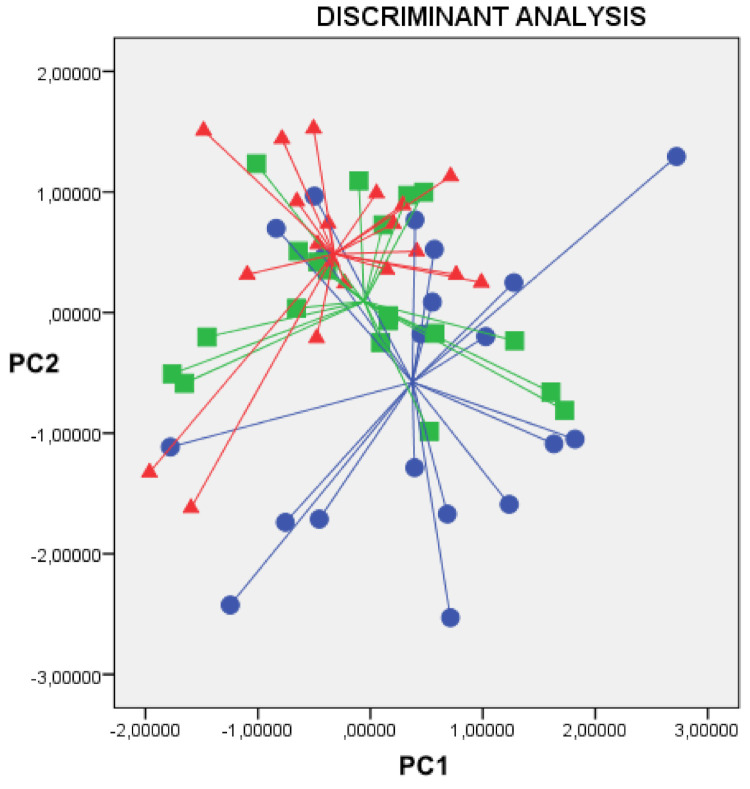
Two-dimensional principal component analysis plot, showing that exhaled VOC profiles among normal ventilation (blue circles), hyperventilation (green squares) and hypoventilation (red triangles) during wash-in are indistinguishable from each other. Cross validated accuracy was 45.1% (*p* = ns). X axis = Principal component 1; Y axis = Principal component 2.

The two-dimensional principal component analysis plot showed that the exhaled VOC profiles obtained for the three breathing rhythms could not be discriminated from each other (Figure 1). The CDA of the data showed a CVA of 45.1%, indicating that the difference was not significant (*p* = ns). Similarly, ANOVA of the main four principal components showed no significant differences among the three groups (*p* = ns for all, see Table 2). Therefore, the Cyranose 320′s 32-sensor outputs from the sensor array were not significantly different among the three breathing rhythms.

## 3. Discussion

According to our results, it appears that the exhaled VOC profile measured by our e-nose is stable during variations in wash-in breathing rhythm.

To the best of our knowledge, this is the first study which specifically investigates e-nose analyzed exhaled breath VOC composition in relation to variation in breathing rhythm in a population of well-characterized, healthy subjects.

Research into the effects of ventilatory variations on exhaled breath composition is essential for a better comprehension of the physiological and metabolic phenotype of healthy subjects, and for implementing exhaled VOC profiling in routine pulmonary medicine.

It is known that a number of VOCs are exhalation flow-dependent, such as acetone, ethanol, pentane and isoprene [10,11,12]. In addition, alterations in exhaled flow, breath hold and dead space significantly modify e-nose assessed exhaled breath patterns with e-nose, thus influencing their ability to discriminate breathprints [13].

Very recently, Sukul et al. analyzed 25 healthy subjects and detected changes in a selection of the most abundant, endogenous and bloodborne VOCs when respiratory rhythms were switched between spontaneous and/or paced breathing [14]. Such changes were closely related to minute ventilation and end-tidal CO2 exhalation [14].

A number of limitations must be taken into account. Firstly, there were a relatively small number of enrolled subjects. However, our sample size with 20 individuals in our proof-of-concept study appeared to be suitable to merit further investigations including larger cohorts and a validation group.

Secondly, although we carefully monitored respiratory rates during sampling, we arbitrarily chose breathing rhythm intervals for each group, and therefore we may have missed some important information.

Thirdly, e-nose analysis does not quantify levels of single VOCs. Incontrovertibly, future studies should incorporate chemical analytical techniques, such as gas chromatography coupled to mass spectrometry (GC-MS) to identify specific discriminant compounds.

How can we explain our results? Human-exhaled breath contains over 3000 VOCs deriving from physiologic and pathophysiological mechanisms, operating via metabolic pathways [15]. In accordance with the findings of previous studies, our results suggest that, although breathing rhythm modifies the individual components of exhaled breath, the overall VOC profile, as measured by an e-nose, does not differ among groups with different breathing rhythms.

What are the implications of our findings? It appears that Cyranose 320 signature patterns output from the 32-sensor array were similar among the three breathing rhythms. Our data indicate that careful breathing rate monitoring during breath collection might not be necessary in future studies using a Cyranose 320. Hence, future research (possibly including patients with functional airways obstruction and restriction) should apply these models into larger clinical trials in order to confirm our findings and to investigate other possible confounding factors. Moreover, these studies must include several types of e-noses, using different technology, in order to assess the interchangeability of devices.

## 4. Materials and Methods

### 4.1. Patients

We enrolled 20 healthy, non-smoking subjects (11 males, 9 females), with a negative anamnesis of chest symptoms and systemic diseases and who were not taking any medications. The age range was 28–55. Lung function was normal for all participants. None of the subjects experienced upper or lower respiratory tract infections in the four weeks before testing, nor during the day of sampling. Subject characteristics are shown in Table 1.

A series of 3 exhaled breath measurements were performed on all subjects, and their exhaled breath was collected at three different breathing rhythms during wash-in phase: (a) normal rhythm (Respiratory Rate (RR) between 12 and 18/min), (b) fast rhythm (RR > 25/min) and (c) slow rhythm (RR < 10/min).

All participants were volunteers and were enrolled from hospital members.

The current study was previously approved by the local ethics committee (protocol number 46403/15) and all participants signed an informed consent before taking part in the study.

### 4.2. Study Design

We performed a longitudinal study. All measurements were obtained during two visits. During the first visit, subjects were carefully checked for inclusion/exclusion criteria and a flow-volume spirometry was performed (MasterscreenPneumo, Jaeger, Wurzburg, Germany).

During the second visit, exhaled breath was collected as described above and immediately analyzed by the e-nose. All participants were randomized to perform a different order of breathing rhythms during the wash-in phase: a-b-c, a-c-b, c-b-a, c-a-b, b-a-c, b-c-a. Intervals between each measurement were at least 2 h. Subjects were asked to refrain from eating and drinking, as well as from engaging in vigorous physical exercise, for at least 3 h before visit two. Breath was collected as follows: first a wash-in phase of 5 min through a 3-way non-rebreathing valve connected to an inspiratory VOC filter (A2; North Safety, Middelburg, The Netherlands) to reduce the effect of environmental VOCs, then subjects exhaled a single vital capacity volume into a Tedlar bag connected to the e-nose.

### 4.3. Electronic Nose

A commercially available e-nose was used (Cyranose 320, Sensigent, Irwindale, CA, USA). It consists of a nano-composite array of 32 organic polymer sensors. The polymers swell when exposed to VOC combinations, which changes their electrical resistance. Raw data are captured as changes in resistance of each of the 32 sensors in an onboard database, producing a distribution (breathprint) that describes the VOC mixture and that can be used for pattern-recognition algorithms. The operating parameters were as follows: Baseline purge: 30 s (pump speed: low); sampling time: 60 s (pump speed: medium), purging time: 200 s (pump speed: high), total run time: 300 s, temperature 42 °C. Post-run purges between samples: 5 min. In addition, pre-conditioning for the sensor array prior to running samples consisted of a 5 min exposure to the room air to assure stability of sensor outputs, followed by a “blank measurement”, as indicated in the operating instructions manual.

### 4.4. Statistical Analysis

The sample size was estimated based on data deriving from previous studies [16]. The raw data of breath samples were analyzed by SPSS software, version 18.0. The same program was used for the random assignment of breathing sequences. Principal component analysis (PCA) and successive linear canonical discriminant analysis (CDA) were calculated, thus providing the cross-validated accuracy percentage (CVA%), which estimates how accurately a predictive model will perform in practice. Furthermore, ANOVA of the main four principal components (which captured 96.3% of the total variance) was performed among the three breathing rhythms. A *p*-value of <0.05 was considered to be statistically significant.

## Figures and Tables

**Table 1:** Clinical characteristics of the study population.

Parameter	Value
Subjects (n.)	20
M\F (n.)	11\9
Age (y.)	37.1 ± 10.2
FEV1%pred.	103.6 ± 10.7
BMI	25.52 ± 2.4
(ex)-smokers (n.)	0
comorbidities(n.)	0

Values are intended as mean ± SD.

**Table 2:** ANOVA of the main four principal components among the three breathing rhythms.

	Normal Rhythm	Fast Rhythm	Slow Rhythm	*p*
PC1	−0.131 ± 1.045	0.050 ± 0.955	0.081 ± 1.035	0.773
PC2	0.374 ± 1.113	−0.054 ± 0.983	−0.320 ± 0.801	0.084
PC3	0.577 ± 1.178	0.091 ± 0.667	0.485 ± 0.814	0.182
PC4	−0.003 ± 1.162	0.008 ± 1.031	−0.005 ± 0.831	0.999

## Data Availability

Dataset can be available upon reasonable request.
